# Pilot study evaluating intravenous amoxicillin-clavulanate as an alternative to piperacillin-tazobactam for general surgery patients

**DOI:** 10.1017/ash.2023.133

**Published:** 2023-03-03

**Authors:** Sarah A. Drost, Irina Rajakumar, Elissa Rennert-May

**Affiliations:** 1 Pharmacy Department, Alberta Health Services, Calgary, Alberta, Canada; 2 Department of Medicine, Alberta Health Services, Calgary, Alberta, Canada; 3 Departments of Medicine, Community Health Sciences, Microbiology, Immunology and Infectious Diseases, University of Calgary, Calgary, Alberta, Canada

## Abstract

In this point-prevalence survey followed by prospective audit and feedback at 4 tertiary-care hospitals in Calgary, Alberta, Canada, we evaluated whether intravenous amoxicillin-clavulanate may be used as a narrower-spectrum alternative to intravenous piperacillin-tazobactam for patients admitted to general surgery services.

In surgical patients with intra-abdominal infections, recommended first-line empiric treatment includes coverage of aerobic gram-negative bacteria and anaerobic bacteria.^1^ Piperacillin-tazobactam is a broad-spectrum intravenous (IV) β-lactam/β-lactamase inhibitor combination antibiotic with activity against *Pseudomonas aeruginosa* that may be used when broader coverage is warranted, such as for hospital-acquired infections.^2^ However, piperacillin-tazobactam use is often a broader-spectrum agent than is required.^3–6^ Also, IV amoxicillin-clavulanate, an aminopenicillin–β-lactamase inhibitor combination antibiotic with activity against gram-positive (including most *Enterococcus* spp), gram-negative, and anaerobic microorganisms,^7^ became available on the Canadian market in July 2020 and may be used as a narrower spectrum alternative to piperacillin-tazobactam when *Pseudomonas aeruginosa* coverage is not indicated.

In this study, we used a 2-phase approach^8^ to evaluate where IV amoxicillin-clavulanate may be used as an alternative to piperacillin-tazobactam for surgical patients.

## Methods

Phase 1 of this study was performed at 4 tertiary-care centers in Calgary, Alberta, Canada, and phase 2 was performed at 2 of these centers. A zonal microbiology laboratory services all 4 sites, and antibiograms are available online.^9^ Patients admitted to general surgery services who were prescribed IV piperacillin-tazobactam at the time of assessment were included.

Phase 1 of this study was a point-prevalence survey. Observational data were collected monthly from September to December 2021. A computer-generated list identified patients admitted to general surgery services who had an active order for IV piperacillin-tazobactam. Patients on piperacillin-tazobactam were evaluated to assess whether therapy change to IV amoxicillin-clavulanate or other alternative therapy would be appropriate based on prespecified criteria developed from institutional and clinical guidelines.

In phase 2 of this study, we implemented a pilot test of prospective audit and feedback (PAF) from January to May 2022. A list of patients admitted to general surgery and prescribed piperacillin-tazobactam was generated once a week. Electronic patient charts were reviewed and a suggestion was made to switch to IV amoxicillin-clavulanate if deemed appropriate. If IV amoxicillin-clavulanate was not the most appropriate alternative agent, other antimicrobial(s) could be suggested. Other antimicrobial stewardship (AMS) interventions could be suggested at this time, and >1 recommendation could be made per AMS intervention. Notes were written in the electronic medical record, but the patient’s medical team could be paged.

We evaluated 3 primary outcomes: (1) percentage of piperacillin-tazobactam orders concordant with current clinical guidelines and AMS practices (phases 1 and 2); (2) percentage of piperacillin-tazobactam appropriate to switch to IV amoxicillin-clavulanate (phases 1 and 2); and (3) percentage acceptance rate of suggestions (phase 2).

Secondary outcomes of interest included percentage of piperacillin-tazobactam orders that would be appropriate to switch to alternate therapy and types of AMS recommendations made.

Data collection was performed via electronic medical record at the time of assessment, consistent with previously established AMS practice at our institution. Because this was a quality improvement project and the AMS review and interventions performed were part of usual AMS activities, ethics review board approval was not required.

## Results

In phase 1, 52 orders for IV piperacillin-tazobactam were screened. Among them, 25 (48%) were categorized as opportunities for AMS optimization. Intravenous amoxicillin-clavulanate was deemed the most appropriate alternate therapy in 2 (8%) of 25 cases. Baseline characteristics of patients from phase 1 are presented in Table 1.

**Table 1.** Phase 1 Patient Characteristics

Patient Characteristic	Total (n=52)
Age, median y (IQR)	66 (53–74)
Sex, male, no. (%)	29 (56)
CCI score, median (IQR)	3 (2–5)
**Type of surgery, no. (%)**	
Emergency	29 (56)
Elective	9 (17)
Nonsurgical	14 (27)
**Anatomical site of infection, no. (%)**	
Intra-abdominal	45 (87)
Liver	1 (2)
Blood	2 (4)
Lungs	9 (17)
Other	1 (2)
Positive culture, no. (%)	13 (25)
Days of antibiotic therapy, median (IQR)	4.5 (2–7)
Had source control procedure, no. (%)	21 (40)
ID service consult, no. (%)	2 (4)

Note. IQR, interquartile range; CCI, Charlson comorbidity index; ID, infectious disease.

**Fig. 1.**
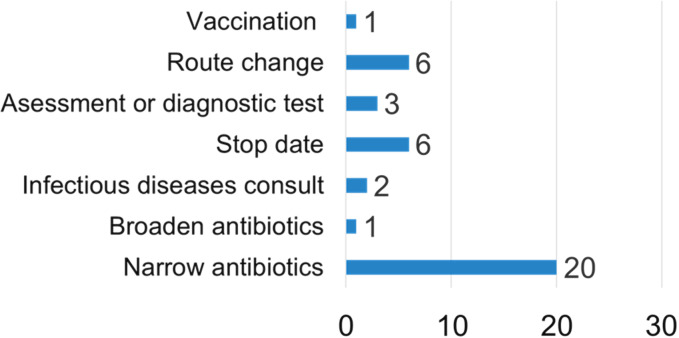
Type of antimicrobial stewardship recommendation.

During phase 2 of the study, 104 IV piperacillin-tazobactam orders were reviewed and 28 (27%) were categorized as opportunities for AMS optimization. In 12 cases, IV amoxicillin-clavulanate was deemed the most appropriate alternate therapy, and the suggestion was accepted in 1 case (8%). Other types of AMS suggestions are presented in Figure 1. Most recommendations were either fully (12 of 28, 43%) or partially (8 of 28, 29%) accepted. Recommendations were considered partially accepted if multiple suggestions were made simultaneously and at least 1 recommendation was not implemented or if only a portion of a single recommendation was accepted.

## Discussion

In this study, we used a 2-phased approach to assess whether IV amoxicillin-clavulanate might present a narrower spectrum alternative to IV piperacillin-tazobactam for general surgery inpatients. Our rate of inappropriate piperacillin-tazobactam prescribing was similar to other studies.^3,5^ To our knowledge, this study is the first to evaluate recommending the use of IV amoxicillin-clavulanate as part of a PAF strategy to decrease piperacillin-tazobactam use.

We present several considerations for recommending IV amoxicillin-clavulanate as a narrower-spectrum alternative to piperacillin-tazobactam. *Pseudomonas aeruginosa* is a pathogen responsible for many hospital-acquired infections and has high rates of resistance to antibiotics.^10^ As expected, piperacillin-tazobactam use is associated with piperacillin-tazobactam–resistant strains of *P. aeruginosa*.^2^ Because *P. aeruginosa* coverage is not indicated in many community-acquired infections, including community-acquired intra-abdominal infections,^1^ using IV amoxicillin-clavulanate (which does not cover *P. aeruginosa*) is a strategy implemented to “preserve” piperacillin-tazobactam antipseudomonal activity. At our institution, Enterobacterales susceptibility to amoxicillin-clavulanate is very similar to piperacillin-tazobactam and ceftriaxone.^9^ Also, IV amoxicillin-clavulanate maintains coverage of *Enterococcus* spp (whereas a cephalosporin and metronidazole combination does not), which may be warranted in specific circumstances.

We primarily evaluated the use of the IV (vs oral) amoxicillin-clavulanate for several reasons. First, the use of oral amoxicillin-clavulanate as step-down therapy for intra-abdominal infections is a well-established practice both at our institution and elsewhere.^1^ We specifically evaluated the AMS niche for a new product. In addition, we anticipated that much of the IV piperacillin-tazobactam use captured during this study would occur shortly after initiation, given the high turnover of this patient population.

Several reasons may explain why uptake of IV amoxicillin-clavulanate suggestions was low. Primarily, IV amoxicillin-clavulanate is new to both the Canadian market and is a recent addition (January 2021) to our formulary. Prescribers may not have been aware of its availability and place in practice. Also, IV administration is different than oral dosing for amoxicillin-clavulanate, so practitioner unfamiliarity may have played a role. In several cases, the surgical team chose to switch patients to oral amoxicillin-clavulanate instead of IV administration. A delay between the patient assessment by the AMS team and the surgical team reviewing the recommendation may have contributed to this finding.

This study had several limitations. The PAF portion of the study took place over 16 weeks, which may not have been an adequate amount of time to observe the uptake of suggestions. Second, we only completed PAF of patients on piperacillin-tazobactam once per week, and the median days of antibiotic therapy was 4.5 for the patients reviewed during phase 1. More frequent PAF would have allowed earlier intervention and would potentially have increased the uptake of IV amoxicillin-clavulanate. Third, although the general surgery department was informed about the PAF phase of the project, no targeted education was given to prescribers, which may have been another way to target piperacillin-tazobactam use. In addition, we did not collect information on whether infections were community or hospital acquired, which may have affected suggestion uptake, although this information was factored into AMS recommendations. Finally, this was a pilot quality-improvement study, and we did not assess clinical outcomes of interest, such as antimicrobial resistance or complications of broad-spectrum antibiotics.

Although we intended to evaluate the use of IV amoxicillin-clavulanate, it is clear that AMS interventions to optimize piperacillin-tazobactam use on general surgery units are needed and that IV amoxicillin-clavulanate is unlikely to have a substantial role as an alternative antimicrobial choice. Future studies evaluating strategies to address piperacillin-tazobactam prescribing on general surgery units are warranted.
